# Clinical outcomes of a novel porcine small intestinal submucosa patch for full-thickness hand skin defects: a retrospective investigation

**DOI:** 10.1186/s13018-023-03531-z

**Published:** 2023-01-17

**Authors:** Chengwu Zang, Hang Xian, Hang Zhang, Min Che, Yongxiang Chen, Fanliang Zhang, Rui Cong

**Affiliations:** 1 Department of Hand Surgery, Xijing Hospital, The Air Force Medical University, Xi’an, 710032 People’s Republic of China; 2 Department of Orthopaedics, Affiliated Central Hospital of Shenyang Medical College, Shenyang, 110020 People’s Republic of China

**Keywords:** Hand, Full-thickness skin defect, Small intestinal submucosa patch, Biomaterial, Hand surgery

## Abstract

**Objective:**

To investigate the clinical outcomes of a novel soft tissue repair patch (porcine small intestinal submucosa patch, SIS patch) in the treatment of full-thickness hand skin defects.

**Methods:**

From January 2017 to July 2019, 80 patients with hand soft tissue defects, who met the inclusion criteria, were retrospectively reviewed and divided into two groups. After debridement, patients in group A were treated with the novel SIS patch to cover the wound, and patients in group B were treated with autologous skin graft. The dimensions of skin defect area and healing outcome were evaluated and recorded. Scar assessment was carried out using Scar Cosmesis Assessment and Rating Scale (SCAR scale) at the last follow-up postoperation, and the recovery of wound sensation was assessed at the same time using British Medical Research Council (BMRC) grading of sensorimotor recovery. All the data were collected and statistically analyzed.

**Results:**

A total of 80 patients were enrolled in the study with 40 patients in each group. Four patients in group A and 5 patients in group B were excluded due to wound infection and lost to follow-up. There were 36 patients in group A and 35 patients in group B finally got follow-up postoperation with mean interval of 12.75 ± 5.61 months in group A and 14.11 ± 5.42 months in group B. The dimensions of skin defect area in group A ranged from 7.5 to 87.5 cm^2^ (mean 25.97 ± 18.66 cm^2^) and in group B ranged from 7.5 to 86.25 cm^2^ (mean 33.61 ± 19.27 cm^2^) which have no significant difference (*P* > 0.05). SCAR scale results of group A and group B were 10.98 ± 0.33 and 9.49 ± 0.35, respectively, and the difference was statistically significant (*P* < 0.05). BMRC grading results showed 6 cases of S4, 11 cases of S3+, 5 cases of S3, 6 cases of S2, 6 cases of S1 and 2 cases of S0 in group A, and 8 cases of S4, 10 cases of S3+, 7 cases of S3, 4 cases of S2, 5 cases of S1, and 1 case of S0 in group B, which had no significant difference between them (*P* > 0.05).

**Conclusions:**

The novel SIS patch is an applicable biological material in the treatment of hand skin defect, which could achieve a better cosmetic appearance of the newborn skin tissue.

## Introduction

The injury of hand always brings devastating impact to patient’s life, which could not only have a serious effect on the function but also on the aesthetic appearance of hands. As hand is one of the most frequently used extremity when manipulating machines, high rate of injury that caused by various injury mechanisms makes it more vulnerable [1, 2]. Soft tissue defects of the hand require systematized methods to restore the function and aesthetic appearance [3, 4]. As stable skin supply and soft tissue coverage are regarded as the primary goal during the reconstruction of mutilated hands, essential consideration on these aspects during surgical planning is getting more and more attention [5]. Several methods including autologous skin grafts and flaps are used to treat full-thickness skin defects of the hand, while contour defect, color mismatch, scar contracture, and donor site complications are still thorn issues during reconstruction procedures [6]. The task of finding an ideal cover tissue which could meet both the aesthetic and treatment needs is still challenging for surgeons and researchers.

This novel porcine small intestinal submucosa patch (SIS patch), which obtained from the decellularized extracellular matrix (ECM) of pig small intestinal submucosa, contains a natural three-dimensional framework composed of collagen I, III, and IV and also retains a variety of growth factors (TGF-β, FGF-2, and CTGF), glycoproteins, proteoglycans, and glycosaminoglycans [7, 8]. The microscopic three-dimensional structure of the collagen framework also could provide a suitable scaffold for the ingrowth of cells, facilitating cell migration to the interior of the implant [9]. These biological characteristics make it commonly used in reconstruction procedures on different fields [10]. Whether this kind of SIS patch is effective in the treatment of full-thickness hand skin defects in clinical work still needs further investigations.

We conducted this retrospective controlled investigation to test the clinical outcomes of the novel SIS patch (Biodesign® Soft Tissue Graft, Cook Biotech Incorporated, West Lafayette, IN) in contrast to traditional autologous skin graft.

## Materials and methods

### Subjects

This retrospective controlled investigation was conducted between January 2017 and July 2019 in the single university affiliated hospital of the Air Force Medical University. The study protocol was approved by the institutional review board, and written informed consent was obtained from all study participants (adult patients and guardians of juveniles). The inclusion criteria were full-thickness hand skin defects. The exclusion criteria were multiple soft tissue defects (such as tendon, nerve, and bone tissue), polytrauma (such as fractures, burn), severe metabolic diseases (such as diabetes mellitus, rheumatic arthritis, and hemophilia), blood disorders, and autoimmune diseases. To achieve good interobserver reliability, certain experienced hand surgeon, who was blinded to the treatment protocol, conducted the Scar Cosmesis Assessment and Rating Scale (SCAR scale) to evaluate the reconstructed skin at the last follow-up postoperation and the British Medical Research Council (BMRC) grading to assess the wound sensation at the same time. A premeasurement standardization session was conducted to evaluate intra-rater reliability before scoring.

**Fig. 1:**
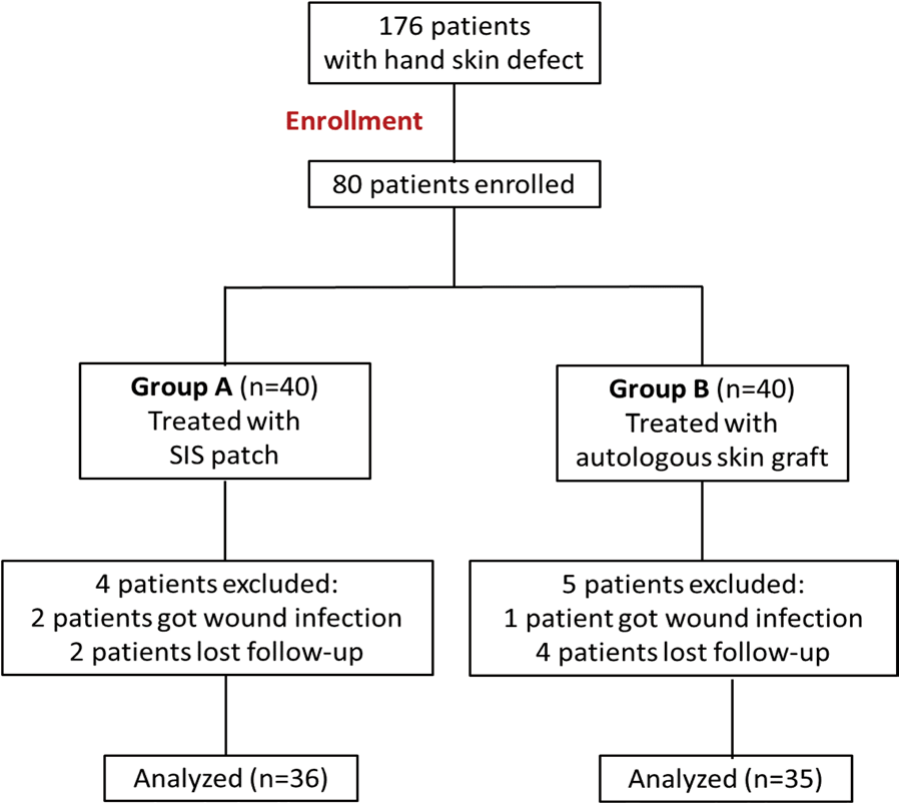
Distribution of the study subjects from enrollment to the end of the study

Patients who fulfilled the inclusion criteria were assigned into 2 groups following different treatment methods: the novel SIS patch to cover the wound group (group A) and autologous skin graft group (group B). Statistics of 176 patients were reviewed, and 80 patients with hand soft tissue defects, who met the inclusion criteria, were enrolled and divided into 2 groups, with 40 patients in each. Four patients in group A and 5 patients in group B were excluded due to wound infection and lost to follow-up. The remaining 71 patients were finally enrolled in the study with 36 patients in group A and 35 patients in group B (Fig. 1). All the surgeries were carried out by a same surgical team. The STROBE guidelines were followed during the whole study design.

### Surgical procedure

Surgical procedures were performed under regional anesthesia. Wounds were managed with debridement and careful trimming of the skin defect edge to remove all devitalized tissue and contaminants until the wound was clean. After carefully debridement, the wound was then repeatedly washed by sterile saline and any active hemorrhage was stopped avoid using electrocoagulation. The dimensions of the skin defects were then measured using a sterilized Vernier caliper. After making sure there is no defect of deep tissue, such as tendon or bone, SIS patch which met the defect size was selected. The patch was submerged in room-temperature, sterile saline for 1–2 min. After the patch was completely hydrated, it was placed on the wound and was secured in place with 5–0 coated Vicryl® Plus antibacterial absorbable suture (Ethicon, Inc.). In group B, autologous graft of full-thick skin was selected to cover the wound and sutured through the similar method as previously reported [11]. Vaseline® gauze was then placed in touch with the patch or skin graft, and sterile wound dressing was used to apply moderate pressure over the entire wound area, keeping the SIS patch or skin graft in intimate contact with the underlying wound bed.

### Postoperative management

The affected hand was immobilized with a brace in a functional position. Oral antibiotics, cefuroxime tablets 125–250 mg, *b.i.d.*, were given for 3 days postoperation. In Group A, the wound dressing was changed every 2 weeks. In group B, the pressure dressings were removed 7–9 days after skin grafting. Functional rehabilitation was gradually started 2 weeks after operation when the sutures were removed. Regular follow-up was performed after treatment for all patients.

### Assessment of treatment effects

Wound healing of all patients was assessed by attending physicians who comprehensively evaluated the therapeutic effects of the treatment. At the last follow-up time postoperation, scar assessment was carried out using Scar Cosmesis Assessment and Rating Scale (SCAR scale) [12] and the recovery of wound sensation was assessed at the same time using British Medical Research Council (BMRC) grading [13] of sensorimotor recovery.

### Statistical analysis

Statistical analysis was performed using SPSS 20.0 for Windows. Nonparametric Mann–Whitney *U* tests were chosen to compare the postoperative subjective outcome scores, and numerical values were expressed and mean and standard deviation, while nominal outcomes were reported as frequency. Chi-square test was used for nominal data of gender, hand involved, and BMRC scale. Any *P* values less than 0.05 were considered statistically significant.

## Results

The details of the baseline of patient characteristics are shown in Table 1, and there is no significant difference found in the baseline characteristics of patients including age, gender, and side of the affected limb. Four patients in group A and 5 patients in group B were excluded due to wound infection and lost to follow-up. There were 36 patients in group A and 35 patients in group B left finally. The postoperation follow-up interval ranged from 4 to 25 months in group A within a mean time of 12.75 ± 5.61 months, and the follow-up time in group B ranged from 6 to 26 months within a mean time of 14.11 ± 5.42 months (*P* > 0.05, Table 1). The dimensions of skin defect area in group A ranged from 7.5 to 87.5 cm^2^ (mean 25.97 ± 18.66 cm^2^) and in group B range from 7.5 to 86.25 cm^2^ (mean 33.61 ± 19.27 cm^2^) which have no significant difference (*P* > 0.05, Table 1). SCAR scale results of group A and group B showed 10.98 ± 0.33 and 9.49 ± 0.35, respectively, and the difference was statistically significant (*P* < 0.05, Table 1). BMRC grading results of showed 6 cases of S4, 11 cases of S3+, 5 cases of S3, 6 cases of S2, 6 cases of S1 and 2 cases of S0 in group A, and 8 cases of S4, 10 cases of S3+, 7 cases of S3, 4 cases of S2, 5 cases of S1, and 1 case of S0 in group B, which had no significant difference between them (*P* > 0.05, Table 1).

**Table 1:** Baseline of patient characteristics and follow-up results

Characteristics	Group A (n = 36)	Group B (n = 35)	*P* value
Mean age, y (range)	23.86 ± 14.82 (5–57)	30.69 ± 16.73 (3–61)	0.0730
Female/male, n	10/26	12/23	0.6140
Right/left hand involved	21/15	24/11	0.4620
Dimensions of defect area, cm^2^ (range)	25.97 ± 18.66 (7.5–87.5)	33.61 ± 19.27 (7.5–86.25)	0.0937
Follow-up, ms (range)	12.75 ± 5.61 (4–25)	14.11 ± 5.42 (6–26)	0.0310
SCAR scale (range)	10.89 ± 2.25 (7–14)	9.49 ± 2.05 (6–14)	0.0077
BMRC grade			0.9160
S4	6	8	
S3+	11	10	
S3	5	7	
S2	6	4	
S1	6	5	
S0	2	1	

**Fig. 2:**
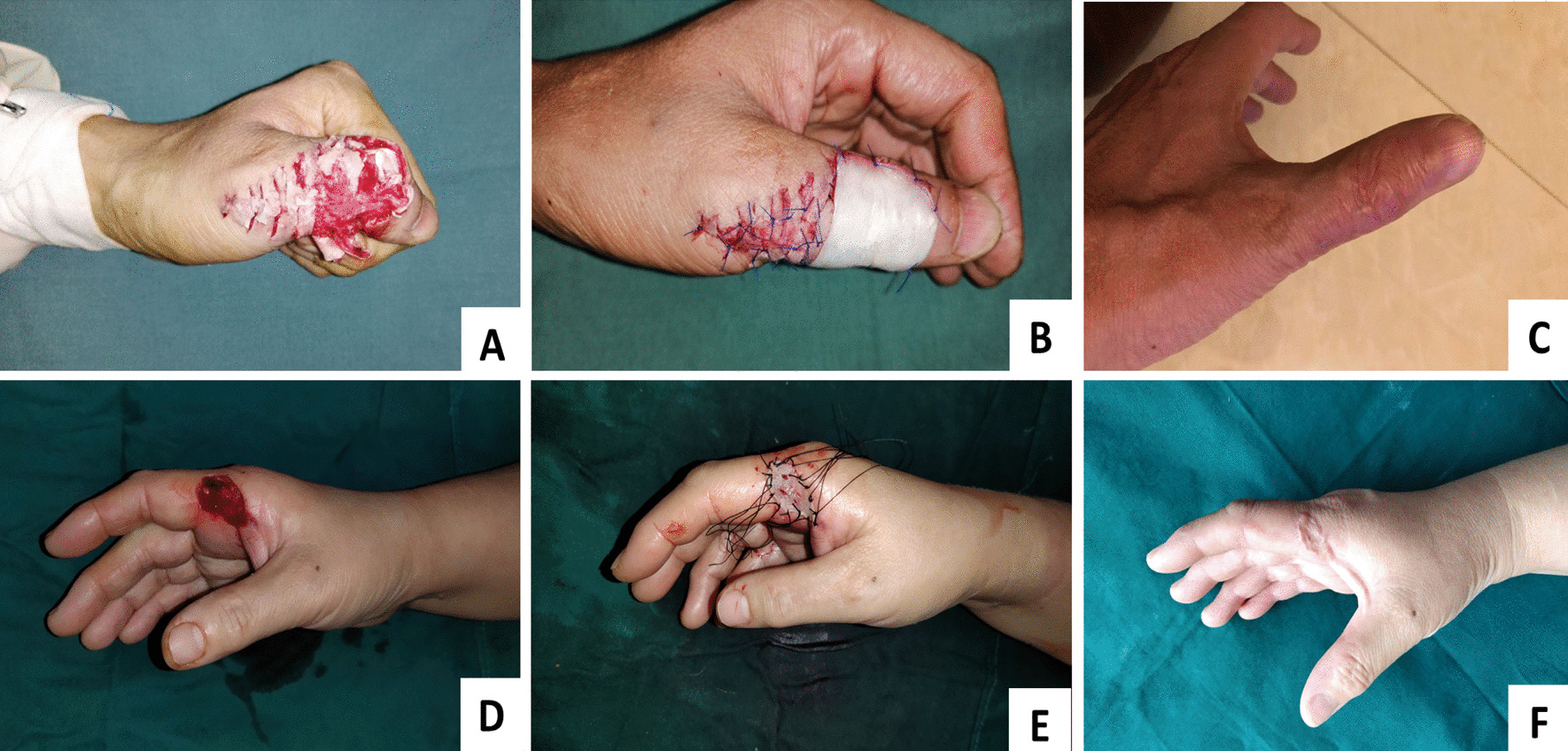
Representative images of two cases in group A (**A**–**C**) and group B (**D**–**F**). **A**–**C** A 51-year-old male farmer suffered a machine twist injury that resulted in severe skin defect of the dorsal part of his left thumb. **A** Appearance of the affected hand after debridement. Exposure of thumb extensor tendon could be observed with a full-thick skin defects about 3.5 cm × 5.0 cm. **B** SIS patch with a suitable size was used to cover the skin defect area, and the edge was sutured to the wound around. **C** Appearance of the affected hand during the last time follow-up. The result of the SCAR scale was 13 and the sensation of the newborn skin was S4 according to the BMRC grading system. **D**–**F** A 28-year-old male worker suffered a sharp injury that resulted in skin defect of the radial part of his right first metacarpophalangeal joint. **D** Appearance of the affected hand after debridement. A full-thick skin defects about 2.0 cm × 3.0 cm could be observed. **E** Autologous full-thick skin graft was selected to cover the defect area. **F** Appearance of the affected hand during the last time follow-up, and texture mismatch and scar hyperplasia could be observed. The result of the SCAR scale was 8 and the sensation of the newborn skin was S3 according to the BMRC grading system

Typical cases are shown in Fig. 2.

## Discussion

Skin defects of the hand due to trauma are common injuries which are often accompanied by exposure of tendon, bone, nerve, and blood vessels [4]. The treatment methods vary widely due to different range of possible injuries which should be individualized considered both to the patients and to the injuries [14, 15]. Typical treatment of skin defects of hand is focusing on autologous skin grafts and flaps, while this kind of traditional treatment methods cannot overcome the vital demerit of the second injury to the donor side where the skin or graft harvested and other demerits including color mismatch, contour and volume mismatch, and texture mismatch, etc. [6]. Moreover, the consequent sensorimotor function recovery of both the donor and the recipient areas is still a problem that not only affects the treatment effect but also brings up the associated burden to the patients and family.

The search for an ideal “synthetic” or “artificial” skin graft, which could be found without using skin tissues from elsewhere in the body to avoid donor-site morbidity during reconstructing the soft tissue defect, is still a formidable task for surgeons [3]. In 1981, O’Connor et al. [16–18] first reported the successful use of autologous cultured human epithelium in the repair of the wounds of 2 burn patients. Subsequent animal-derived artificial skin, Integra® Matrix Wound Dressing (Integra Life Sciences, Plainsboro, NJ), was successfully developed as a skin substitute to promote the repair of damaged tissue. With rapid development of biological tissue engineering technologies, tissue engineering of skin now employs one of three distinct approaches including tissue constructed and cultured in vitro using seeded cells and scaffold materials, tissue composed of cells only, and tissue engineering of scaffold materials to promote epithelialization [19–21]. Meanwhile, xenogeneic tissues, which are not limited by the autologous source required of current approaches, are becoming a focal area of research.

The ideal artificial skin coverage should be easy to obtain, convenient to use, bioactive, and protective. Since SIS patch has been used more than 3 decades in the field of medicine, it has been shown to help tissue regeneration and angiogenesis, such as in skull base reconstruction [22, 23], upper airway tissue remodeling [24], tympanic membrane repair [25], cardiovascular diseases [26]. The natural three-dimensional ECM framework composed of collagen I, III, and IV and a variety of bioactive substances it contains makes it helpful during wound healing [7, 8], and this kind of particular composition enables it rapid the rate of angiogenesis and does not activate immune system [9, 10]. The natural three-dimensional structure of the collagen framework could facilitate cell migration, and cells in the wound bed could secrete endogenous regulatory factors, which can stimulate the growth of new tissue, regulate immune function, and transport stem cells to the affected area at the same time [10]. This kind of crosstalk makes adequate preparations for the healing of the wound. Meanwhile, the ECM could protect these growth factors from inactivation and prolong their half-life [27], which enables the biological factors and polysaccharides in the ECM keep playing their roles in the affected area, including promoting tissue regeneration, vascular growth, and neuronal differentiation [28]. The SIS patch has been reported useful in musculoskeletal reconstructions of the foot and ankle due to its characteristics of augmentation effect on cellular and vascular in-growth [29], while another report of Hodde et al. [30] showed SIS patch did not improve the rate of tendon healing or the clinical outcome scores. The treatment effect of SIS patch in glenohumeral arthritis is also ambiguous [31]. Whether SIS patch is superiorly useful in hand skin reconstruction is still unclear. Our group had used SIS patch to cure hand skin defects, and the preliminary effects seems satisfying. Here, we conducted this retrospective investigation to report the results.

The initial principal aims of any type soft tissue reconstruction are to restore the form, function, and sensation of the hand [6]. Color mismatch, contour and volume mismatch, texture mismatch, donor-recipient tissue interface, hair growth and the following scar location and development of skin contractures, and overall appearance of the donor site are all issues that need to be considered preoperation. The novel soft tissue repair patch used in this study can supply adequate strength to be sutured and is also soft enough to cover the wound bed to provide a barrier against the outside pathogen environment [32, 33]. The last follow-up results in this study show that this novel SIS patch is really helpful in skin defect that it could supply stable coverage until the new growth of the skin tissue. Further evaluation of the newborn skin shows that the SCAR scale of the novel patch group is superior to the autologous skin graft group, while the BMRC grading of sensation assessment has no significant difference between them. These results may indicate that in situ regeneration could better mimic the surrounding affected skin during wound healing, which may be the ideal of skin regeneration process. It is inseparable that the coverage of the novel SIS patch could supply a perfect microenvironment during this process. In the meantime, without sacrifice of other skin tissue of the body could be treated as another advantage of the novel SIS patch, which could eliminate the corresponding risk of the donor side, while the financial burden of the patients and society of patch used should be a potential issue to be reconsidered. The sensation recovery of the new skin tissue still needs further research to be verified.

This study also presents some limitations. First, the sample size of the current study is small, and larger number of patients and prospective study are needed to support the advantages of the novel SIS patch and perfect the indications. This trial includes adults and children could be treated as another limitation, as many potential differences may exist between them. Further research that can differentiate this issue is needed in the future to confirm the results. Meanwhile, advanced research on the mechanisms of the novel SIS patch during tissue recovery is also needed to explore more evidence on the fueling tissue growth. Last, we focused on the treatment of full-thickness hand skin defects, which may be an underlying limitation of the study. As hand injuries always accompanied by multiple tissue injuries (such as bone or nerve tissue), whether this novel SIS patch is still effective in this complex tissue reconstruction procedure must be further validated, which could be the direction of future study.

## Conclusions

Soft tissue defect of the hand is still a thorny issue for surgeons, and many limitations of autologous skin graft are also unavoidable. The novel SIS patch could supply a stable coverage of the wound for full-thickness skin defects patients and achieve a better cosmetic appearance of the newborn skin tissue. This novel patch could be an ideal biomaterial in the reconstruction surgery of patients with skin defects, especially for the ones with single full-thickness skin defects. Further prospective study on the mechanisms is still needed to explore its other merits.
